# Fructose consumption induces molecular adaptations involving thyroid function and thyroid-related genes in brown adipose tissue in rats

**DOI:** 10.1590/1414-431X2022e12240

**Published:** 2023-01-16

**Authors:** J.G.O. Neto, J.S. Romão, C.C. Pazos-Moura, K.J. Oliveira

**Affiliations:** 1 Departamento de Fisiologia e Farmacologia, Universidade Federal Fluminense, Niterói, RJ, Brasil; 2 Instituto de Biofísica Carlos Chagas Filho, Universidade Federal do Rio de Janeiro, Rio de Janeiro, RJ, Brasil

**Keywords:** Thyroid hormone receptor, Deiodinase, Lipid metabolism, Metabolic dysfunction, Thermogenesis

## Abstract

The increasing incidence of metabolic diseases is in part due to the high fructose consumption, a carbohydrate vastly used in industry, with a potent lipogenic capacity. Thyroid hormones (TH) are essential for metabolism regulation and are associated with changes in body weight, energy expenditure, insulin sensitivity, and dyslipidemia. This study aimed to investigate the influence of fructose intake on thyroid function and thyroid-related genes. Male Wistar rats were divided into Control (CT, n=8) and Fructose (FT - 10% in drinking water, n=8) groups for three weeks. The FT group showed higher glycemia and serum triacylglycerol, indicating metabolic disturbances, and increased thyroid mass, accompanied by higher expression of *Srebf1c* and *Lpl*, suggesting increased lipid synthesis. The FT group also presented higher expression of *Tpo* and *Dio1* in the thyroid, suggesting activation of the thyroid gland, but with no alterations in serum TH concentrations. Brown adipose tissue (BAT) of the FT group exhibited higher expression of *Dio2*, *Thra*, and *Thrb*, indicating increased T3 intra-tissue bioavailability and signaling. These responses were accompanied by increased BAT mass and higher expression of *Adrb3*, *Pparg*, *Srebf1c*, *Fasn*, *Ppara*, and *Ucp1*, suggesting increased BAT adrenergic sensitivity, lipid synthesis, oxidation, and thermogenesis. Therefore, short-term fructose consumption induced thyroid molecular alterations and increased BAT expression of thyroid hormone-related signaling genes that potentially contributed to higher BAT activity.

## Introduction

The increasing worldwide incidence of metabolic diseases such as obesity, insulin resistance, dyslipidemia, and non-alcoholic fatty liver disease (NAFLD) has been associated with the high consumption of fructose (1). The adverse metabolic effects of fructose may lead to ectopic lipid accumulation in metabolic tissues, such as liver and muscle (2), and a dysfunctional lipid accumulation in brown adipose tissue (BAT), which compromises its thermogenic capacity (3).

Thyroid hormones (TH) are essential for development, growth, and metabolism, and thyroid dysfunction is associated with changes in body mass, energy expenditure, insulin sensitivity, and dyslipidemia (4,5). The thyroid gland produces triiodothyronine (T3) and thyroxine (T4) hormones, regulated mainly by pituitary thyroid-stimulating hormone (TSH), which stimulates TH biosynthesis and release. Peripheral metabolism is mediated by the action of deiodinases, essential enzymes for the conversion of T4 to T3, the biologically active hormone. Thyroid hormone effects in target tissues are mainly mediated by the genomic control of gene expression induced by T3 interaction with the nuclear thyroid hormone receptors (THR) (6).

Nutrition directly impacts thyroid physiology. A high-fat diet (HFD) disrupts the thyroid lipid profile (7) and leads to abnormal morphology of the gland (8), and a short- and long-term high carbohydrate diet affects thyroid axis activity and alters serum hormone concentrations in animals (9,10) and in humans (11,12).

Therefore, we hypothesized that short-term fructose consumption disrupts thyroid hormone production and impacts thyroidal action and BAT metabolism. In this study, we demonstrated that fructose intake led to altered thyroid homeostasis, modifying the expression of genes related to hormonogenesis and lipogenesis in the thyroid gland, and genes associated with TH metabolism and action in the BAT.

## Material and Methods

### Ethical approval

Experimental procedures were approved by the Ethics Committee on Animal Use of the Fluminense Federal University (protocol #757/2016). The study followed the Animal Research Reporting *in Vivo* Experiments (ARRIVE) guidelines and complied with the ethical guidelines of the Brazilian Society of Laboratory Animal Science.

### Experimental design

Adult male Wistar rats (∼2 months of age) provided by the Fluminense Federal University animal facility were kept in a temperature-controlled room (23±1°C) with artificial light-dark cycles (12/12 h, lights on at 7 am). Sixteen rats were randomly divided into 2 groups: the control group (CT, n=8) and the fructose group (FT, n=8). The FT group received D-fructose (Sigma Aldrich, USA) diluted in drinking water (10%) for 3 weeks. The animals were housed in collective polyethylene cages (four animals per unit), receiving commercial chow (Nuvilab Cr-1^®^, Nuvital Nutrientes S/A, Brazil) and water *ad libitum*. The 10% dosage of D-fructose used is efficient to induce the metabolic syndrome phenotype in rats (13) and resembles the daily fructose consumption described in humans (14). Body mass gain and food intake were measured every 3 days and water intake was measured daily throughout the experiment. The animals were sacrificed by decapitation (after a 3-h fast). Glucose was measured from the blood of the trunk using a glucometer (ACCU-Chek Advantage, Roche, Germany). The serum was separated by centrifugation (15 min, 4°C, 1600 *g*) and stored at -70°C. The pituitary was harvested. BAT (interscapular depot), visceral white adipose tissue (epididymal and retroperitoneal depots), and thyroid were dissected and weighed.

### Serum measurements

Serum triacylglycerol was measured by colorimetric assays using commercial kits (Labtest, Brazil). Triglyceride-glucose (TyG) index is strongly associated with type 2 diabetes development and was calculated following the equation: Ln [fasting triacylglycerol level (mg/dL) × fasting glucose level (mg/dL) / 2] (15).

Serum total T3 (TT3), total T4 (TT4), and free T4 (FT4) were measured using solid-phase radioimmunoassay kits (Linco Research, USA). Intra-assay variation was 2.57% for TT3, 5.11% for TT4, and 3.32% for FT4.

### Real-time PCR

Total RNA from BAT, thyroid, and pituitary was isolated using TRIzol reagent (Invitrogen, USA). cDNA was synthesized using 1 µg of total RNA using the Superscript III kit (Invitrogen). Genes of interest were analyzed by real-time PCR using the GoTaq^®^ qPCR Master Mix (Promega, USA). The oligonucleotide primer sequences for *Thra*, *Thrb*, *Dio1*, *Dio2*, *Dio3*, *Tshb*, *Tpo*, *Srebf1c*, *Fasn*, *Lpl*, *Ppara*, *Pparg*, *Ppargc1a*, *Ucp1*, *Adrb3*, and *Rplp0* are reported in Supplementary Table S1. Relative mRNA expression levels (2^-ΔΔCt^) were calculated after correction for the reference gene *Rplp0* (36β4). The fructose group was compared to the control group, which was considered to be 1. The purity of the PCR products was assessed by melting curve analysis.

### Statistical analysis

Data normality was verified by the Shapiro-Wilk test. Statistical analysis was performed using Student's *t*-test or Mann-Whitney test using GraphPad Prism 6 software (version 6.01, USA). Data are reported as means±SE and statistical differences were considered significant at P<0.05.

## Results

The consumption of 10% fructose for three weeks led to no changes in final body mass (P=0.821), body mass (BM) gain (P=0.993), and visceral fat mass (absolute mass, P=0.073; mass corrected by BM, P=0.174). However, fructose induced higher liver mass (absolute mass, P=0.001; mass corrected by BM, P=0.002), glycemia (P=0.009), serum triacylglycerol (P=0.015), and TyG index (P=0.042) (Table 1).

**Table 1:** Metabolic parameters of animals of the control group and fructose group after 3 weeks of treatment.

Parameters	Control group	Fructose group	P value
Body mass (g)	382.3±11.45	385.8±9.99	0.821
Body mass gain (g)	36.74±6.46	36.67±3.90	0.993
Visceral fat mass (g)	7.47±0.69	9.12±0.94	0.073
Visceral fat mass/body mass	0.026±0.001	0.024±0.002	0.174
Liver mass (g)	12.52±0.46	14.57±0.60*	0.001
Liver mass/body mass	0.032±0.0007	0.037±0.001*	0.002
Glycemia (mg/dL)	92.60±1.60	100.8±1.65*	0.009
Serum triacylglycerol (mg/dL)	103.00±15.29	213.10±38.38*	0.015
Triglyceride-glucose index	4.52±0.09	4.95±0.13*	0.031
Daily water intake (mL/animal)	33.56±2.8	76.80±8.0*	0.007
Daily fructose caloric intake (Kcal/animal)	-	31.67	-
Daily chow intake (g/animal)	24.65±1.43	18.33±0.51*	0.014
Daily total caloric intake (Kcal/animal)	82.83±4.83	93.27±2.69	0.132
Thyroid mass (g)	1.58±0.08	2.15±0.20*	0.016
Thyroid mass/body mass (mg/g)	0.041±0.001	0.056±0.006*	0.039
Brown adipose tissue mass (g)	0.33±0.02	0.47±0.03*	0.004
Brown adipose tissue mass/body mass (mg/g)	0.89±0.06	1.25±0.10*	0.009

Data are reported as means±SE. *P<0.05, Student's *t*-test.

Daily water intake was higher in the FT group (P=0.007), but chow intake by the FT group was lower (P=0.014), with no differences in caloric intake (Table 1).

**Figure 1:**
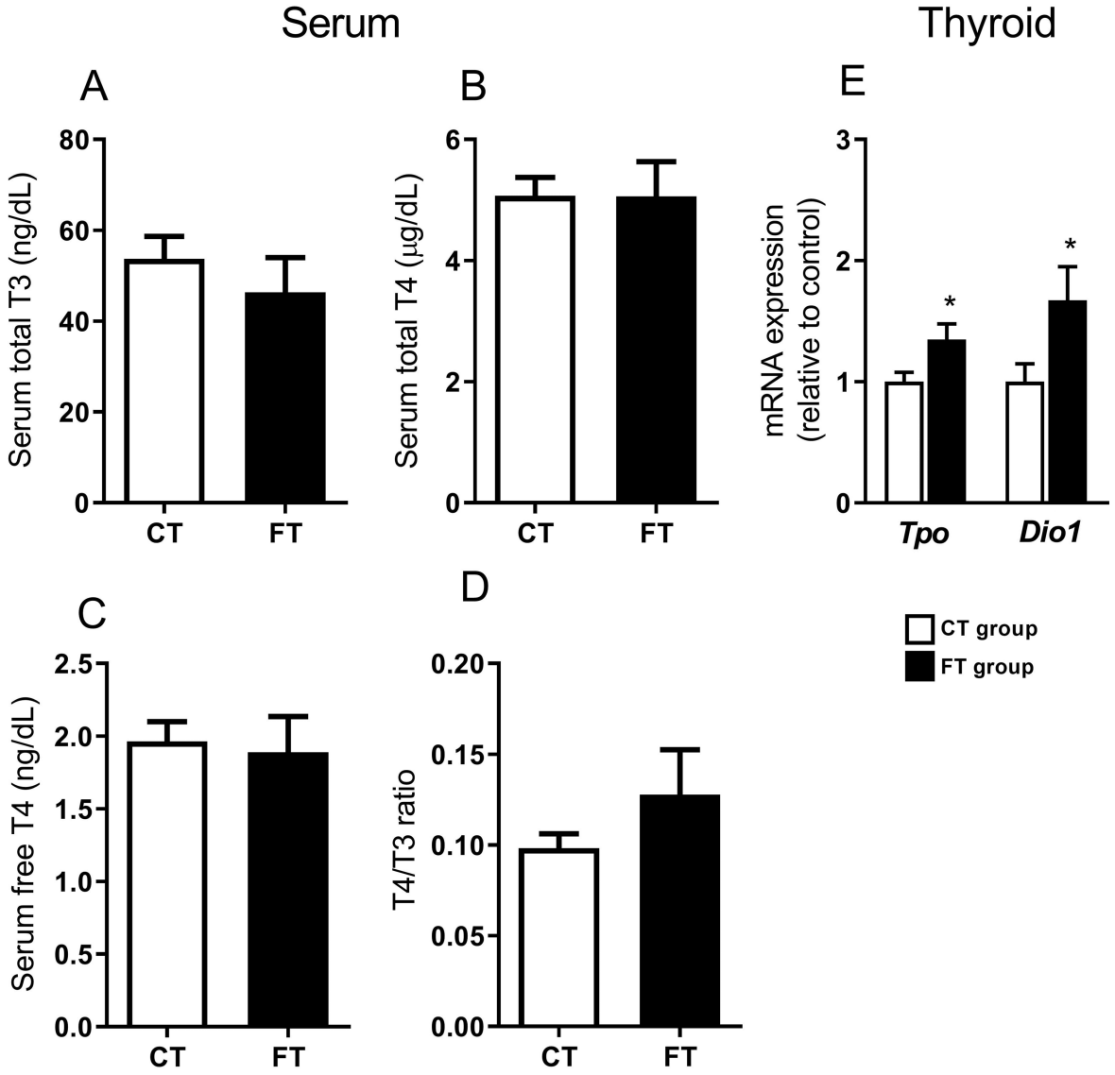
Serum concentrations of thyroid hormones and thyroid mRNA expression. **A**, Serum total T3. **B**, Serum total T4. **C**, Serum-free T4. **D**, T4/T3 ratio. **E**, Expression of thyroperoxidase (*Tpo*) and deiodinase type 1 (*Dio1*) in the thyroid. The real-time PCR results are corrected by the reference gene *Rplp0* and expressed relative to the values of the control group, which was set to 1. Control group (CT); Fructose group (FT - 10% fructose diluted in drinking water, for 3 weeks). Data are reported as means±SE (6-8/group). *P<0.05, Student's *t*-test.

No alterations in serum TT3 (total T3), TT4 (total T4), FT4 (free T4), or T4/T3 ratio were observed in the FT group (Figure 1A-D). However, the FT group presented higher thyroid mass (absolute mass, P=0.016; mass corrected by BM, P=0.039; Table 1), accompanied by higher mRNA expression of *Tpo* (thyroperoxidase - TPO) (P=0.031), and *Dio1* (deiodinase type 1 - D1) (P=0.044) (Figure 1E) compared to the CT group.

**Figure 2:**
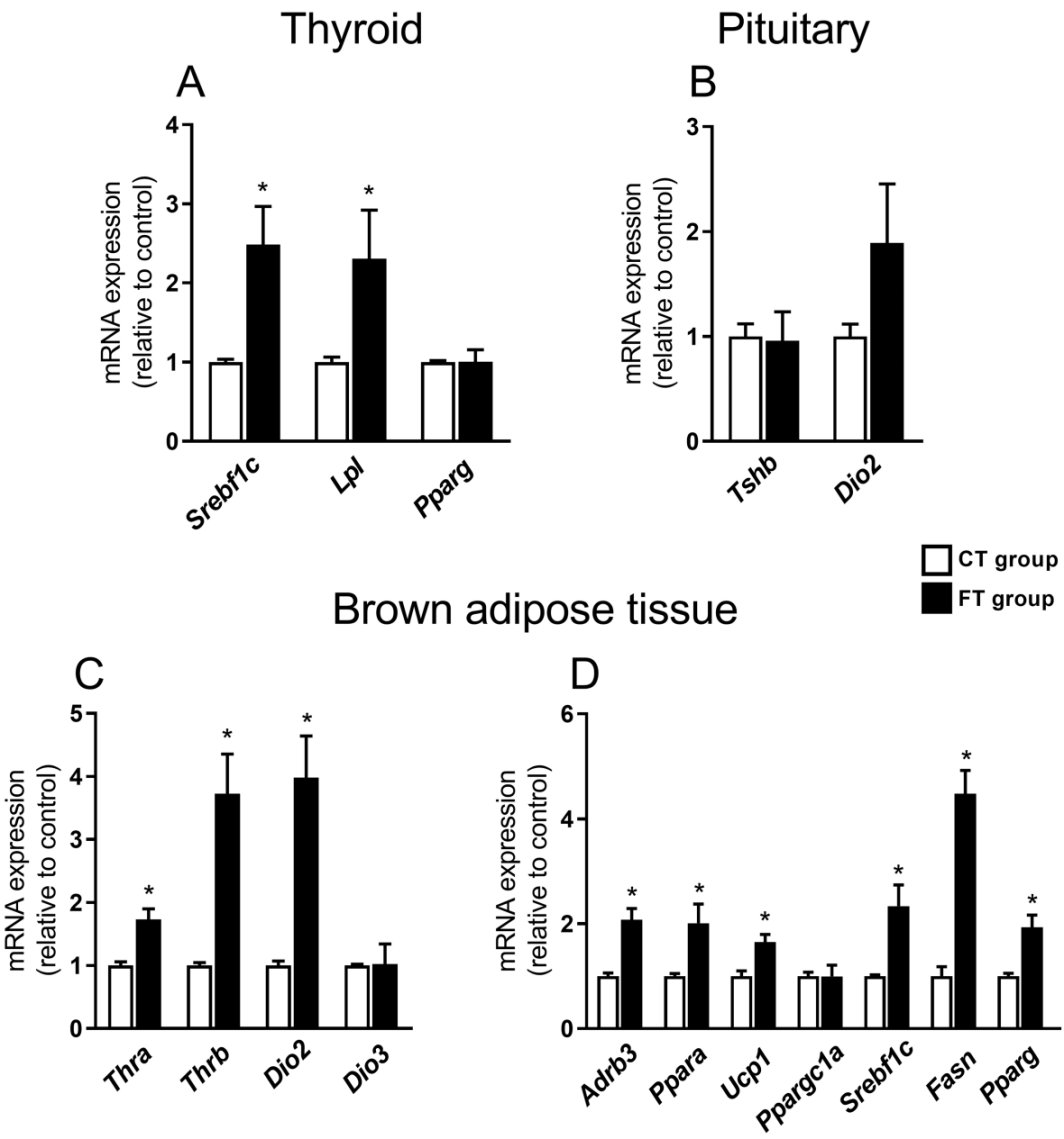
Molecular analysis of the thyroid, pituitary, and brown adipose tissue. **A**, Expression of sterol regulatory element-binding protein-1c (*Srebf1c*), lipoprotein lipase (*Lpl*), and peroxisome proliferator-activated receptor isoform gamma (*Pparg*) in the thyroid. **B**, Expression of thyroid-stimulating hormone isoform beta (*Tshb*) and deiodinase type 2 (*Dio2*) in the pituitary. **C**, Expression of thyroid hormone receptor isoform alpha (*Thra*), thyroid hormone receptor isoform beta (*Thrb*), deiodinase type 2 (*Dio2*) and deiodinase type 3 (*Dio3*) in the brown adipose tissue. **D**, Expression of adrenergic receptor beta 3 (*Adrb3*), peroxisome proliferator-activated receptor isoform alpha (*Ppara*), uncoupling protein 1 (*Ucp1*), peroxisome proliferator-activated receptor gamma coactivator 1 alpha (*Ppargc1a*), sterol regulatory element-binding protein-1c (*Srebf1c*), fatty acid synthase (*Fasn*), and peroxisome proliferator-activated receptor isoform gamma (*Pparg*) in the brown adipose tissue. The real-time PCR results were corrected by the reference gene *Rplp0* and expressed relative to the values of the control group, which was set to 1. Control group (CT); Fructose group (FT - 10% fructose diluted in drinking water, for 3 weeks). Data are reported as means±SE (6-8 per group). *P<0.05, Student's *t*-test.

Thyroid gland of the FT group showed higher mRNA expression of *Srebf1c* (sterol regulatory element-binding protein-1c - SREBP1c) (P=0.0058) and *Lpl* (lipoprotein lipase) (P=0.0403), with no change in the *Pparg* (peroxisome proliferator-activated receptor gamma - PPARγ) expression (Figure 2A).

No significant differences were observed in the pituitary expression of *Tshb* (thyroid-stimulating hormone isoform beta - TSHβ) and *Dio2* (deiodinase type 2 - D2) (Figure 2B).

The FT group had higher BAT mass (absolute mass, P=0.004; mass corrected by BM, P=0.009; Table 1), associated with higher expression of *Thra* (P=0.0009), *Thrb* (P=0.0004), *Dio2* (P=0.0002) (Figure 2C), *Adrb3* (adrenergic receptor beta 3 - ADRβ3) (P=0.0002), *Ppara* (peroxisome proliferator-activated receptor alpha - PPARα) (P=0.011), *Ucp1* (uncoupling protein 1 - UCP1) (P=0.002), *Srebf1c* (P=0.003), *Fasn* (fatty acid synthase - FAS) (P=0.0007), and *Pparg* (P=0.001) (Figure 2D).

No alterations were detected in the *Dio3* (deiodinase type 3 - D3) (Figure 2C) and *Ppargc1a* (peroxisome proliferator-activated receptor gamma coactivator 1 alpha - PGC1α) expressions (Figure 2D).

## Discussion

The present study showed that short-term fructose consumption induced metabolic disturbances and altered thyroid homeostasis by modifying the expression of genes associated with hormonal biosynthesis in the thyroid gland and genes associated with TH metabolism and action in BAT.

Fructose consumption for three weeks led to higher liver mass, glycemia, serum triacylglycerol, and TyG index, indicating a metabolic syndrome phenotype as observed in humans and in animal models (1,16-18). The higher serum triacylglycerol and glucose levels, and the increased TyG index in the FT group suggested insulin resistance development (15,19), even without changing body mass gain and visceral adiposity. In addition, hypertriglyceridemia induced by fructose or HFD consumption has been associated with lipid accumulation and/or lipotoxicity in the BAT (3,19) and thyroid (20).

As expected, daily water intake was higher in the FT group (17). However, as a compensatory mechanism, the chow intake by the FT group was lower, maintaining a similar caloric intake.

No alterations in TT3, TT4, FT4, or TT4/TT3 ratio were observed in the FT group, indicating that the fructose overload for 3 weeks did not disrupt TH serum concentration. However, short-term fructose consumption induced higher thyroid mass, accompanied by higher mRNA expression of *Tpo*, a key enzyme for TH biosynthesis (6), and *Dio1*, an enzyme that metabolizes TH (6), suggesting increased thyroid activity. This may be regarded as a compensatory mechanism for the higher metabolic demand from the fructose overload. In early stages of excessive fructose consumption, higher TPO activity may be necessary for maintaining normal serum TH levels. Others have shown evidence that the fructose impact on thyroid hormonogenesis is time-dependent (9). Five weeks of fructose consumption induced higher thyroid iodine uptake, despite reduced serum TT3 and FT3 in rats. After ten weeks of fructose, TT4 was reduced accompanied by lower iodine uptake. These data may indicate that high fructose intake by individuals with clinical and subclinical hypothyroidism may worsen their clinical condition.

Ectopic lipid accumulation is one of the adverse metabolic effects induced by fructose intake (21,22). We showed that the FT group had increased expression of lipogenic markers, such as *Srebf1c* and *Lpl* in the thyroid gland. Obesity and/or dyslipidemia have been associated with tissue lipotoxicity, as well as structural and functional changes in the thyroid gland that were associated with impairment in TH synthesis (23) and hypothyroidism (7,23). Our data suggested that fructose potentially leads to lipid accumulation that could result in lipotoxicity in the thyroid, as reported in other tissues under fructose overload (16,17).

The thyroid gland is under the control of TSH, responsible for stimulating TH synthesis and release and controlling thyrocyte growth and proliferation (6). The lack of alteration in the pituitary expression of *Tshb* may reflect normal levels of serum TSH. However, the expression of genes related to TH synthesis can be controlled by other regulators, such as SREBP1c (24,25). Therefore, we speculated that the changes in the mRNA expression of thyroid genes and/or thyroid mass induced by fructose intake could be due to the increased expression of *Srebf1c*, a secondary response to dyslipidemia, insulin resistance, or a direct effect of fructose (26).

BAT thermogenesis induced by noradrenaline (NE) and T3 involves the participation of transcription factors such as PPARα (involved in fatty acid oxidation) and PGC1α (a central inducer of mitochondrial biogenesis), as well as UCP1, which allows heat production in the mitochondria inner membrane (27). Therefore, the higher expression of *Adrb3*, *Ppara*, and *Ucp1* in the fructose-treated animals could be a consequence of the higher expression of *Thra* and *Thrb* in the FT group. Furthermore, THRα activation by T3 is associated with higher ADRβ3 expression and action (28). As a result, the higher *Thra* expression observed in fructose-treated rats could contribute to increasing *Adrb3* signaling, potentializing the NE effects in the BAT. The higher *Dio2* mRNA expression observed in the FT group may be associated with increased NE signaling in BAT, which is known to stimulate *Dio2* gene transcription (28). D2 is localized in the endoplasmic reticulum membrane and catalyzes the bioactivation of T4, ensuring T3 bioavailability (6). The deletion of the *Dio2* gene leads to deficient thermogenic capacity, lower lipogenesis, and fatty acid oxidation, highlighting the importance of intracellular T3 for these mechanisms (29,30). Therefore, increased levels of *Dio2* accompanied by higher THRs mRNAs expression should contribute to the higher expression of UCP1 and further increased thermogenesis induced by fructose intake. The BAT activation observed in the FT group could contribute to preventing the increase in visceral adipose tissue mass and body mass gain during the short-term fructose overload.

Brown adipose tissue *de novo* lipogenesis seems to be stimulated in fructose-treated animals, once a higher expression of *Srebf1c*, *Fasn*, and *Pparg* were observed in the FT group. Higher levels of fatty acid could be used as an important energy source (31), which is in line with the higher expression of genes related to fat oxidation and thermogenesis in the FT group. Reports have shown that T3 induces lipid synthesis in the BAT (29,32), therefore, the increased expression of *Dio2*, *Thra*, and *Thrb* could contribute to the higher expression of lipogenic markers induced by short-term fructose intake.

Chronic high-fructose diets have been associated with reduced respiratory quotient, indicating increased catabolism of fat and a reduction in metabolic rate (9). Therefore, the increased lipid synthesis and oxidation observed in animals with short-term high fructose consumption highlights that the metabolic and thermogenic adaptation are temporally different and may be stimulated by the local increase in the TH signaling pathway in the BAT.

Therefore, the short-term high fructose consumption induced thyroid alterations without changing TH serum concentration. Moreover, fructose promoted molecular adaptation in the BAT suggesting higher T3 intra-tissue bioavailability and signaling. This thyroid-related adaptation in BAT seems to contribute, at least in part, to a higher adrenergic sensitivity and increased lipid synthesis, oxidation, and thermogenesis in response to fructose overload. The study highlighted the negative metabolic impact of excessive fructose intake, including hyperglycemia and hypertriglyceridemia.

## Supplementary Material

Click here to view [pdf].
